# Liberia—Moving Beyond “Ebola Free”

**DOI:** 10.3201/eid2111.151322

**Published:** 2015-11

**Authors:** Hunter Keys, John Midturi, Laura Chambers-Kersch

**Affiliations:** University of Amsterdam–Amsterdam Institute for Social Science Research, Amsterdam, the Netherlands (H. Keys);; Baylor Scott & White Healthcare, Temple, Texas, USA; (J. Midturi);; University of New Mexico, Albuquerque, New Mexico, USA

**Keywords:** Ebola, Ebola virus infection, Liberia, West Africa, social justice, viruses

Although the ongoing Ebola epidemic has brought much attention to Liberia, diseases of poverty, such as malaria, tuberculosis (TB), and maternal–newborn complications, rarely make the headlines. Along with the other West African countries that bore the brunt of the epidemic, Liberia ranks near the bottom of the Human Development Index, a composite measure that assesses whether persons enjoy a long and healthy life, can acquire knowledge, and have an adequate standard of living (*1*). In Liberia, before the Ebola outbreak, ≈50 doctors attempted to care for ≈4 million persons (*2*). In an already fragile health-care setting, Ebola took a terrible toll: >8% of the health care workforce in Liberia died from the virus (*3*). The consequences of such a dramatic loss will be felt for years to come, especially in the areas of infectious disease and maternal and infant mortality (*3*). As we renew our commitment to make Liberia “Ebola free,” we should remind ourselves that in the 21st century, Liberians still die from 19th century diseases. The focus must go beyond “getting to zero.” As concerned clinicians, we argue that much more work needs to be done.

In early 2015, we went to Grand Gedeh County as short-term clinicians, working with a nongovernmental organization (NGO) to support Liberia’s existing health care infrastructure. Unlike emergency response NGOs, our NGO turned its attention to assisting local hospitals, clinics, and communities in their routine, day-to-day health care activities. Tucked away in Liberia’s remote southeastern corner, Grand Gedeh County had largely been spared from the epidemic; at that point, only 1 case had been reported since the epidemic’s onset. We would not wear the protective space suits so familiar in the public eye. Instead, we would work in surrounding communities, meeting with local health workers and psychosocial officers, or on the wards with Liberian nurses and doctors, tending to persons who suffered, and at times died, from easily preventable diseases.

The public hospital offered a glimpse into the state of Liberian health care facilities in the wake of Ebola. The building itself lacked electricity most hours of the day; it had no running water, and there was a severe shortage of medications and basic supplies. The only available pain medication at the hospital was oral acetaminophen tablets. We witnessed 5 neonatal deaths in 10 days. In the community, we listened to first-hand experiences about the peak of the epidemic from those who had relocated to Grand Gedeh County. They recounted how entire families died in the span of weeks and how fear and stigma rent communities apart. Nonetheless, working alongside and learning from our colleagues and friends filled us with deep admiration and humility. Despite their country’s history of war, poverty, and disease, the Liberians we met believe they can create something better.

Indeed, the inner strength and commitment of our Liberian colleagues prompted much self-reflection. In the throes of the epidemic, Liberian health care workers provided care at the expense of their own safety, and many died. Even before Ebola, however, these same health care workers were risking their own health and well-being, and that of their families, by treating patients with TB without respiratory masks or going without basic vaccinations—all fundamental, common-place measures taken in American hospitals. Our contribution as short-term health care providers seemed minuscule compared with the reality that our Liberian colleagues continue to face. This personal struggle was made all the more disturbing by knowing that an exit was already planned for us. Our Liberian colleagues and friends remain.

As we reflect on our brief time in Liberia, we revisit the central issue that compelled us to go: do we, in our position of comfort and relative ease, have a responsibility to help impoverished persons who have an exotic and frightening disease? We still emphatically believe that the answer is yes. But shouldn’t we go further and ask whether that responsibility includes diseases and deaths that have become routine, accepted, or rendered invisible? 

The story of Ebola confirmed that we are all connected in a complex sphere of exchange—of resources, technologies, ideas—and that the arrangement benefits some more than others. It was not coincidental that so many persons in that part of the world fell ill from the virus. Decades of conflict, economic impoverishment, and a near-nonexistent health care system created a tangle of injustices that set the stage for the epidemic. We share the conviction that these injustices—unfair social, political, and economic arrangements—are the real culprits behind the Ebola epidemic and, in broader terms, today’s health disparities. Our participation reminds us that the Ebola experience is but one example of how politics and poverty cause death and suffering on a grand scale.

We should examine how the Ebola epidemic, and the global response to it, fit into this larger picture of health disparities and injustice. Ebola linked Monrovia, Liberia, to Dallas, Texas, USA, and in so doing exposed how interconnected our global society has become. While infected international health workers were evacuated to specialized centers with experimental drugs, infected Africans hoped for supportive care in crowded Ebola treatment units, circumstances that forced us to grapple with the unfairness of today’s health care inequities. Despite years of bureaucratic research, financing, and planning, the epidemic’s unprecedented scale demanded major reexamination of what is meant by the concept of global preparedness. Thus, in terms of understanding how health disparities should be addressed, Ebola overturned key assumptions: that rich countries can ensure the health of their populations in isolation, that fundamental ethical issues regarding the role of global health agencies and their actors are settled, and that a single, global authority can marshal and coordinate resources effectively (*4*).

Why don’t we apply the same lessons to diseases of poverty and other emerging infectious diseases? De Cock and colleagues note, “It is difficult to explain why investment in separating human drinking water from human feces, the basis of the nineteenth century public health revolution in Europe and North America, has not been a higher political or development priority in resource-poor settings” (*5*, p. 1195). If we acknowledge our interconnectedness through social, economic, and political dimensions, that there exist severe shortcomings in health equity both within and between countries, and that these challenges require cooperation beyond the traditional donor-recipient model, then perhaps we will come to terms with why deaths from malaria, TB, or diarrheal diseases implicate all of us, and matter (*5*). Engagement in global health is not just a humanitarian endeavor; it is a priority for our collective well-being.

As the Ebola crisis wanes and media attention shifts away from West Africa, the underlying determinants of health and disease are still in place. The reemergence of Ebola virus infection attests to this, as do the senseless deaths of those who die from easily preventable diseases. We must hold ourselves to a higher standard, one beyond a 42-day Ebola-free countdown. With self-reflection and in a spirit of solidarity, we must continue to articulate that standard and where our responsibility lies in meeting it.
